# Targeting gallbladder cancer: oncolytic virotherapy with myxoma virus is enhanced by rapamycin *in vitro* and further improved by hyaluronan *in vivo*

**DOI:** 10.1186/1476-4598-13-82

**Published:** 2014-04-13

**Authors:** Mingzhe Weng, Wei Gong, Mingzhe Ma, Bingfeng Chu, Yiyu Qin, Mingdi Zhang, Xueqing Lun, Grant McFadden, Peter Forsyth, Yong Yang, Zhiwei Quan

**Affiliations:** 1 Department of General Surgery, Xinhua Hospital, Shanghai Jiaotong University, School of Medicine, Shanghai 200092, China; 2 Department of Oncology, Southern Alberta Cancer Research Institute, University of Calgary, Calgary, Alberta, Canada; 3 Molecular Genetics and Microbiology, University of Florida, Gainesville, Florida 32610, USA; 4 Department of Neuro-oncology, H. Lee Moffitt Cancer Center & Research Institute, Tampa, Florida 33612, USA

**Keywords:** Gallbladder cancer, Myxoma virus, Oncolytic virotherapy, Collagen IV

## Abstract

**Background:**

Gallbladder carcinoma (GBC) is highly lethal, and effective treatment will require synergistic anti-tumor management. The study is aimed at investigating the oncolytic value of myxoma virus (MYXV) infection against GBC and optimizing MYXV oncolytic efficiency.

**Methods:**

We examined the permissiveness of GBC cell lines to MYXV infection and compared the effects of MYXV on cell viability among GBC and control permissive glioma cells *in vitro* and *in vivo* after MYXV + rapamycin (Rap) treatment, which is known to enhance cell permissiveness to MYXV by upregulating p-Akt levels. We also assessed MYXV + hyaluronan (HA) therapy efficiency by examinating Akt activation status, MMP-9 expression, cell viability, and collagen distribution. We further compared hydraulic conductivity, tumor area, and survival of tumor-bearing mice between the MYXV + Rap and MYXV + HA therapeutic regimens.

**Results:**

MYXV + Rap treatment could considerably increase the oncolytic ability of MYXV against GBC cell lines *in vitro* but not against GBC xenografts *in vivo*. We found higher levels of collagen IV in GBC tumors than in glioma tumors. Diffusion analysis demonstrated that collagen IV could physically hinder MYXV intratumoral distribution. HA–CD44 interplay was found to activate the Akt signaling pathway, which increases oncolytic rates. HA was also found to enhance the MMP-9 secretion, which contributes to collagen IV degradation.

**Conclusions:**

Unlike MYXV + Rap, MYXV + HA therapy significantly enhanced the anti-tumor effects of MYXV *in vivo* and prolonged survival of GBC tumor-bearing mice. HA may optimize the oncolytic effects of MYXV on GBC *via* the HA–CD44 interaction which can promote viral infection and diffusion.

## Novelty & impact statements

Myxoma virus (MYXV), a rabbit-specific poxvirus, is characterized by its narrow host tropism and efficient tumor killing, which has not been studied in Gallbladder carcinoma (GBC), the most common biliary tract malignancy featured by its high lethality, aggressive nature, and dismal prognosis. Here, we found MYXV + Rap treatment could considerably increase the oncolytic ability of MYXV against GBC cell lines *in vitro* but not against GBC xenografts *in vivo*. It was indicated that extracellular tissue collagen IV hinders MYXV dissemination implanted GBC tumors. Moreover, HA–CD44 interaction may not only elevate viral proliferation by activating Akt but also promote viral spread within GBC tissue by degrading collagen IV through MMP-9 secretion. Our results offer a preclinical rationale for utilizing MYXV as a novel therapeutic strategy in treating GBC and other tumor with high-expression of collagen.

## Introduction

Gallbladder carcinoma (GBC) remains the most common biliary tract malignancy characterized by its high lethality, aggressive nature, and dismal prognosis [1]. As standard radio- and chemotherapy are insufficient treatments, surgical resection is the only potential curative approach. However, few patients qualify for surgery, leading to a 5% overall 5-year survival rate [2]. Thus, novel therapeutic strategies are needed.

Oncolytic viruses that selectively infect and kill tumors exhibit modest clinical success [3]. Myxoma virus (MYXV), a rabbit-specific poxvirus, exhibits narrow host tropism likely as a consequence of protective induced-interferon (IFN) responses in other species [4]. MYXV can infect and kill over 70% of tested human tumor cell lines by exploiting the same cellular defects such as IFN-mediated mutations [5].

Akt, a serine/threonine kinase important in balancing cell survival, proliferation, and cell death, is dysregulated in many human cancers [6]. Endogenous phosphorylated Akt (p-Akt) levels highly correlate to permissiveness for MYXV infection. Tumor cell lines exhibiting high p-Akt are susceptible to MYXV and defined as type I cells; those with low but detectable p-Akt that increase following MYXV infection are type II; and those with undetectable p-Akt that generally resist MYXV are type III [7]. Rapamycin (Rap), a macrocyclic lactone, increases the oncolytic potential of MYXV by elevating endogenous p-Akt in the context of MYXV infection. Additionally, Rap is an immunosuppressant that modifies host innate or adaptive cellular immunity, further facilitating MYXV infection [8]. Combined MYXV + Rap therapy has successfully treated glioma, medulloblastoma, and other tumors [9,10]. Whether combined therapy can target GBC, however, remains unknown.

Hyaluronan (HA), a large glycosaminoglycan (GAG) [11], is a chief extracellular matrix (ECM) component that contributes significantly to cell proliferation and migration. HA is natively a large polymer but degrades into low-molecular-weight HA under inflammation [12-14]. All CD44 isoforms contain an HA-binding site in their extracellular domain and serve as the major HA cell-surface receptors [15]. HA–CD44 binding stimulates a number of signaling pathways. Among them, firstly, HA activates PI3K/Akt/mTOR signaling [16], which also elevates p-Akt; in the second place, HA induces matrix metalloproteinase-9 (MMP-9, gelatinase B) expression [17]. MMP-9 preferentially degrades denatured collagens and native collagen type IV, a main component of ECM and basal membranes. ECM structures present a barrier to therapeutic molecules and virus particle diffusion within tissues, which may affect the effectiveness of virotherapy [18].

In the present study, we showed that Rap enhanced MYXV-mediated GBC oncolysis *in vitro,* but not *in vivo.* Furthermore, we demonstrated that collagen IV was a critical factor hindering intratumoral MYXV distribution and it limited MYXV-mediated anti-tumor effects *in vivo.* Finally, HA-induced Akt activation and MMP-9 production significantly improved host survival following MYXV + HA therapy.

## Materials and methods

### Cell lines

Three human gallbladder cancer cell lines were used: GBC-SD (Cell Bank of the Chinese Academy of Sciences, Shanghai, China); NOZ (Health Science Research Resources Bank, Osaka, Japan); and SGC-996 (Academy of Life Science, Tongji University, Shanghai, China). CV-1 (monkey kidney), NIH3T3 (murine fibroblast) and U251 (human giloma) cell lines were purchased from the Cell Bank of the Chinese Academy of Sciences. GBC-SD, NOZ, and NIH3T3 cells were cultured in DMEM (Gibco BRL, Carlsbad, CA, USA) containing 15% FBS (HyClone, Logan, UT, USA). SGC-996, CV-1 and U251 cells were cultured in RPMI medium 1640 (Gibco BRL) with 15% FBS at 37°C and 5% CO_2_.

### Virus

The MYXV construct for transfection studies, vMyx-gfp, contains a green fluorescent protein (GFP) cassette driven by a synthetic vaccinia virus early/late promoter [19]. Control UV-inactivated MYXV (termed “dead virus,” or DV) was irradiated for 2 h.

### Reagents

Rat anti-CD44 mAb (clone 020, isotype IgG_2b_) (CMB-TECH, Inc., San Francisco, CA) blocked HA by recognizing the HA-binding region common among all CD44 isoforms. Low-molecular-weight HA (LMW-HA) fragments were purchased from RD (Minneapolis, MN, USA). Rap was obtained from Wyeth Pharmaceuticals, Inc. (Collegeville, PA, USA).

### Viral replication assays

For single-step growth analysis, MYXV at a multiplicity of infection (MOI) of 5 was added to a 95% confluent cell monolayer. After 1 h adsorption, inoculum was removed, and each well was washed 3× with 1× PBS. Supplemented DMEM was added before incubation (37°C). Cells were collected by cell scraping at 1, 4, 8, 12, and 24 h post-infection. Following a 5-min spin (1500 rpm), cells were resuspended in 100 μL of hypotonic swelling buffer. To release virus, each Eppendorf tube underwent 3 freeze–thaw (−80°C and 37°C, respectively) cycles. Lysed cells were sonicated for 1 min and centrifuged (1500 rpm) for 5 min to disaggregate virus complexes.

For multi-step growth analysis, cells were infected (MOI = 0.01) and collected at 12, 24, 48, 72, and 96 h, and infectious virus was titrated in CV-1 cells [20]. Serial virus dilutions (10^−2^ to 10^−8^) in serum-supplemented DMEM were added to CV-1 cells. After viruses adsorbed (1 h), un-adsorbed virus was removed, and DMEM was added to each well. Infection proceeded for 48 h. Titers (FFU/mL) were calculated as the number of foci × dilution × 2. Foci were counted from each well containing <100 foci under the fluorescent microscope (Leica); average titers were calculated from counts obtained from at least two wells.

### Cell viability assays

Cell viability was determined by the water-soluble tetrazolium (WST)-1 method using the WST-1 cell proliferation and cytotoxicity assay kit (Beyotime, Shanghai, China). Briefly, 5 × 10^3^ cells were seeded in 200 μL/well culture medium in 96-well plates for 24 h and treated with Rap or HA for 72 h. After incubation with WST-1 reagent for 2 h at 37°C, absorbance (450 nm) was measured using an automated microplate reader (Bio-Rad 5 Model 550, Bio-Rad, Hercules, CA, USA). Cell viability percentage = mean optical density (OD) of one experimental group/mean OD of the control × 100%.

### Western blotting

Western blot examined protein expression using antibodies against MYXV M-T7 and Serp-1 (Biogen, Cambridge, MA); host p-Akt (Thr308) and Akt (Cell Signaling Technology, MA, USA); and host collagen I and IV (abcam, Cambridge, UK). β-Actin was used as the control. Crude membranes were prepared in lysis buffer (Hepes [10 mM], pH 7.4; NaCl [38 mM]; PMSF [25 μg/mL]; leupeptin [1 μg/mL]; and aprotinin [1 μg/mL]) and centrifuged at 33000 rpm for 1 h, and the pellet was resuspended. Tumor tissues were collected after virus infection, washed with 1× PBS, and subsequently lysed with lysis buffer containing protease inhibitors. Proteins were quantified using the Bradford protein assay (Beyotime, CHN), separated by 10–12% SDS-PAGE, and transferred to PVDF membranes (Millipore, Billerica, MA, USA), which were blocked with 5% non-fat dry milk and incubated with primary antibodies. Proteins were visualized by the ChemiDoc™ XRS image system (Bio-Rad) using the appropriate secondary antibodies conjugated to horseradish peroxidase.

### Real-time PCR

Total RNA was extracted using Trizol (Gibco BRL) according to the manufacturer’s instructions. After quantification, complementary DNA (cDNA) was synthesized from 2 μg of total RNA using a Takara RNA PCR kit (Takara Bio Inc., Dalian, China). Primers were designed by Primer Premier software version 5.0 (PREMIER Biosoft, Palo Alto, CA, USA) and synthesized by Sangon Biotech (Shanghai, China). The following sequences were selected: MMP-9, CGGACCAAGGATACAGTTTGTT (forward) + GCGGTACATAGGGTACATGAGC (reverse); CD44, GAAGATTTGGACAGGACAGGAC (forward) + CGTGTGTGGGTAATGAGAGGTA (reverse). PCR program: initial denaturation at 95°C for 5 min, 40 cycles of 94°C for 20 s and 61°C for 20 s for annealing extension. β-Actin was used as the control.

### Viral diffusion assays

BD Biocoat inserts for 24-well plates were pre-coated with collagen IV on 3-μm membranes (BD Biosciences, San Diego, CA, USA). Briefly, GBCs were plated at the base (1.5 × 10^5^ cells/well). After 24 h incubation, inserts containing vMyx-gfp (MOI = 5) were placed on top. After 24 h, MYXV diffusion was analyzed by calculating the area of fluorescent foci/field in the base using Image-Pro Plus 6.0 software (Media Cybernetics Inc., Washington, USA).

### Histology and immunohistochemistry

Tissues were immediately washed twice with physiologic salt solution followed by fixation in 4% paraformaldehyde for 24 h. After paraffin-embedding, 5-μm serial sections were cut, deparaffinized in xylene, and rehydrated in graded alcohols, followed by 3 rinses with 1× PBS. Antigen retrieval was performed in 10 mmol/L citrate buffer (pH 6.0) at 98°C for 10 min, and the sections cooled to room temperature (20 min). Sections were incubated in 1% H_2_O_2_ for 15 min to block endogenous peroxidase and incubated with 1:100 rabbit polyclonal anti-collagen IV at 4°C overnight. The corresponding biotinylated goat anti-rabbit IgG (Vector, BA-1000) (1:200) was added for 30 min, washed 3× in PBS, and incubated at room temperature in ABC complex (Vectastain ABC kit, Vector Cat# PK-6100) for 30 min. Staining was detected with DAB peroxide substrate solution for 5 min, followed by briefly rinsing in distilled water. Slides were dehydrated in graded ethanol, cleared in xylene, and mounted with Permount medium after counterstaining with Gill’s hematoxylin solution for 3 min. Control sections were incubated with the antibody preincubated with a blocking peptide. Sections omitting primary antibodies were used as negative controls.

### Immunohistochemical scoring system

Immunostained sections were scored by 2 pathologists with no knowledge of experimental details using a semi-quantitative histologic scoring (H-Score) method [21]; contradictory scores were re-evaluated until consensus was reached. Briefly, immunostaining intensity was scored as follows: 0 = none; 1 = weak; 2 = moderate; and 3 = intense compared to strong staining intensity of intratumoral macrophages. The designated H-Score value was obtained by multiplying each intensity (I) with the corresponding percentage of positive areas (PC) [H-Score = ∑(I × PC)]. Final score values ranged from 0–300.

### Transwell invasion assay

Cell migration was evaluated using BD Matrigel Matrix Thin Layer 24-well plates (BD Biosciences). Sub-confluent cells were serum-starved for 24 h before the experiment. Cells were harvested by trypsin/EDTA, washed, resuspended in FBS-free media at a 10^6^ cells/mL density, and transferred (100 μL) onto the matrigel. Lower chambers were filled with 600 μL of media containing 20% FBS, and the plates were incubated at 37°C for 24 h. Transwells were removed, stained with 1% crystal violet, and non-migrating cells were scraped off with a cotton swab. Six fields/Transwell were photographed using an inverted microscope (200×).

### Hydraulic conductivity assay

Tumor-bearing mice were anesthetized by breathing diethyl ether. Evans blue solution (0.04%) was infused into tumor centers with 28G needle connected to a reservoir via 0.52-mm tubing. Infusion pressure (P_inf_) was defined by the reservoir height relative to the needle tip. Flow rate (Q) measured the velocity of the bubble inside the tube. Hydraulic conductivity was based on Darcy’s law for unidirectional flow in an infinite region around a spherical fluid cavity: hydraulic conductivity = Q/(4πa_0_ P_inf_), where a_0_ was the initial fluid-cavity radius that approximately equaled the 28G needle radius (0.18 mm). Here, all hydraulic conductivity was measured under 50 cm H_2_O (P_inf_ = P_50cm H2O_), and all the measurements were repeated 5× in different tumors [22].

### Gelatin zymogram analysis

Gelatinolytic activity was visualized on zymograms as described [23]. Briefly, protein samples (50 μg) were separated on 10% SDS-PAGE containing 1 mg/mL gelatin. Gels were then washed with 50 mM Tris–HCl (pH 7.5) and 2.5% Triton X-100 buffer for 30 min; washed with above buffer plus 5 mM CaCl_2_, and 1 μM ZnCl_2_ for 30 min; and incubated with above buffer plus 10 mM CaCl_2_, and 200 mM NaCl for 24 h (for supernatant) or 48 h (for membrane and tissue extracts) at 37°C. Zymograms were stained with 0.5% Coomassie blue.

### *In vivo* studies in CD-1 nude mice bearing gallbladder cancer cells

Female CD-1 nude mice (age: 5 weeks; weight: 20–25 g) were obtained from the Shanghai Laboratory Animal Center of the Chinese Academy of Sciences (Shanghai, China) and housed at 3–5/cage on a 12-h light/dark schedule at 22 ± 1°C and 50 ± 5% relative humidity. All procedures followed the Ethics Committee guidelines of Xinhua Hospital, School of Medicine, Shanghai Jiaotong University.

Xenograft tumor models were established by subcutaneously injecting GBC-SD, SGC-996, or U251 cells (1 × 10^7^ cells/0.1 mL) into the right flank. Nine days later, vMyx-gfp or DV (1 × 10^7^ PFU) was intravenously injected every other day for a total of 3 injections. For testing Rap, mice were randomly divided (n = 5/group): (a) DV, (b) vMyx-gfp, (c) vMyx-gfp + Rap (5 mg/kg/d injected intraperitoneally beginning 5 days after tumor implantation, continuing 5 times/week for 2 weeks). For testing HA, mice were randomly divided (n = 5/group): (a) DV, (b) vMyx-gfp, (c) vMyx-gfp + Rap, (d) vMyx-gfp + HA (200 μg/mL injected intratumorally at multiple points every other day for 2 weeks beginning 9 days after tumor implantation), (e) vMyx-gfp + HA + anti-CD44 (100 μg/mL). Tumor areas were measured every 3 days. On day 13 after injection, mice were imaged with the Xenogen IVIS Spectrum system to record GFP-labeled virus in tumors, which were then removed for histological examination. For survival studies, animals were followed until sacrifice was required or the experiment was terminated. To examine vMyx-gfp distribution, frozen tumor tissues were cut into 5-μm serial sections, and GFP expression was imaged using a fluorescence microscope. GFP signal intensity was analyzed with ImagePro software to quantify both GFP and total tumor area.

### Statistical analysis

Statistical Analysis Software (SAS Institute, Inc.) analyzed all statistics. Survival curves were generated by the Kaplan-Meier method. All reported P values were two-sided and considered to be statistically significant at *P* < 0.05. All experiments were performed at least 3 times.

## Results

### Myxoma virus infects and kills human gallbladder cancer cell lines *in vitro*

**Figure 1:**
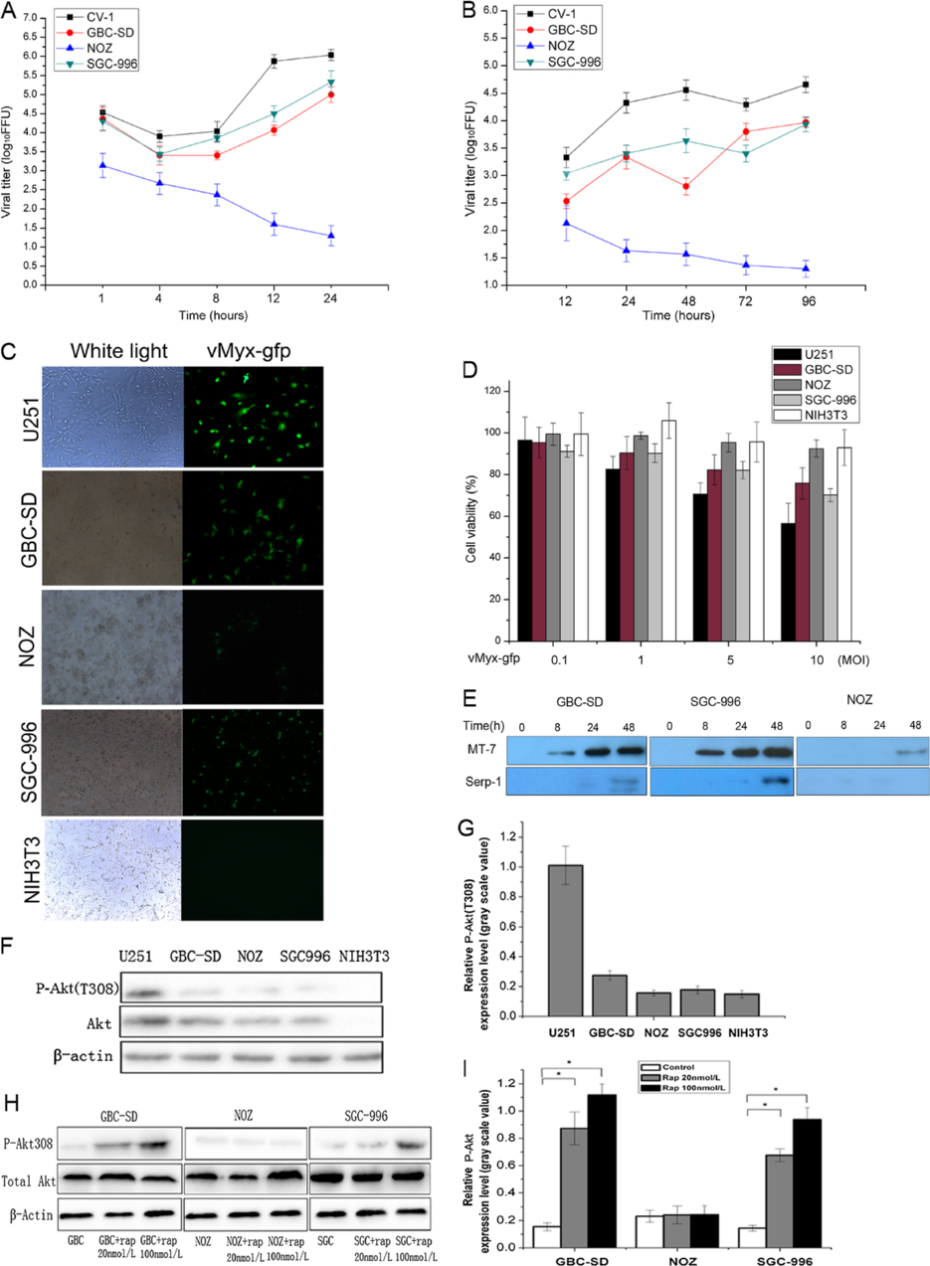
**Myxoma virus productively infects human gallbladder cancer cell lines *in vitro *and phosphorylated Akt expression levels in human gallbladder cancer cell lines. A**. Replication over a period of 1 replication cycle was investigated using high multiplicity of infection (MOI) single-step growth curves in control permissive CV-1, GBC cell lines (GBC-SD, NOZ, SGC-996). All cells were infected with vMyx-gfp (MOI = 5), and cell lysates were collected at the indicated time points after infection. Viral titers were determined by titration in CV-1 cells. **B**. Replication over a period of multiple replication cycles was investigated using low MOI multi-step growth curves. CV-1, GBC-SD, NOZ and SGC-996 were infected with vMyx-gfp (MOI = 0.01). **C**. GFP was in visualized by fluorescence microscopy. GBC-SD, NOZ, SGC-996, a permissive glioma cell line control (U251), and a poorly-permissive murine fibroblast cell line control (NIH3T3) were infected with vMyx-gfp at an MOI = 5 and photographed 48 h after infection. **D**. Effects of MYXV on cell viability of GBC cell lines *in vitro*. **E**. Early viral protein was determined by M-T7 expression, and late viral protein was determined by Serp-1 expression at the indicated time points by western blot of cell lysates. **F**. The expression of phosphorylated Akt (Thr308) in U251, GBC-SD, NOZ, SGC-996, and NIH3T3 cells was evaluated by western blotting. **G**. Densitometry results of relative p-Akt expression normalized by β-Actin of each cell line in Figure 1F. **H**. GBC-SD, SGC-996, and NOZ were pretreated with Rap (20 nmol/L or 100 nmol/L) for 1 h, and then cells were infected with vMyx-gfp (MOI = 5). Levels of p-Akt (Thr308) and total Akt in cell lysates were determined by western blotting. **I**. Densitometry results of relative p-Akt expression normalized by β-Actin of each cell line in Figure 1H. FFU, fluorescent focus-forming units.

To test whether MYXV-based virotherapy could be novel therapeutic against the difficult-to-treat GBC, we first explored the permissiveness of GBC-SD, SGC-996, and NOZ cell lines to vMyx-gfp infection by performing single- and multi-step viral growth curves. While GBC-SD and SGC-996 displayed permissive viral multiplication similar to the permissive CV-1 controls, NOZ cells exhibited a poorly permissive phenotype with a continual decline in titers over 24 hours (Figure 1A). In multi-step growth curves (Figure 1B), viral titers progressively increased in GBC-SD and SGC-996 cells within 96 hours, but CV-1 controls consistently exhibited higher titers. Again, NOZ cells exhibited a pooly permissive phenotype. Monitoring GFP expression at 48 hours post-infection confirmed GBC-SD and SGC-996 permissiveness as well as NOZ poor-permissiveness to infection similar to poorly-permissive control NIH3T3 cells (Figure 1C), and all cells exposed to UV-inactivated DV were GFP-negative (data not shown). Thus, MYXV can infect and replicate in some, but not all, GBC cells.

To determine whether MYXV infection could lead to GBC cell death, we examined cell viability after vMyx-gfp infection utilizing the WST-1 method. MYXV infection killed 24.2% GBC-SD and 29.9% SGC-996 cells, but only 16.4% of NOZ cells (10 MOI, 72 hours); comparatively, 56.6% of control permissive glioma cells (U251) and 92.9% of poorly-permissive NIH3T3 were still metabolically active (Figure 1D). Testing the ability of virus to produce progeny and spread to other cells, western blotting analysis showed that GBC-SD and SGC-996 both expressed MYXV-encoded M-T7 (a protein produced early in the viral life cycle) at 8 hours and Serp-1 (a protein produced late) at 48 hours (MOI = 5), whereas NOZ produced relatively low M-T7 levels even at 48 hours (Figure 1E). Thus, WYXV can successfully replicate in GBC-SD and SGC-996, but not in NOZ.

### Pretreatment with rapamycin enhances viral replication and MYXV oncolysis in GBC cells *in vitro*

Elevated p-Akt levels highly correlate with MYXV permissiveness in some tumors [7]. We studied whether Rap treatment could increase GBC susceptibility to infection by elevating p-Akt levels. Immunoblotting analysis revealed relatively low p-Akt levels in all GBC lines compared to the control permissive U251 glioma cells (Figures 1F,G). Rap treatment (20 or 100 nmol/L) significantly increased p-Akt levels in GBC-SD and SGC-996, but not in NOZ (Figures 1H,I). Therefore, GBC-SD and SGC-996 could be defined as MYXV-permissive type II cells, and NOZ as poorly-permissive type III cells.

**Figure 2:**
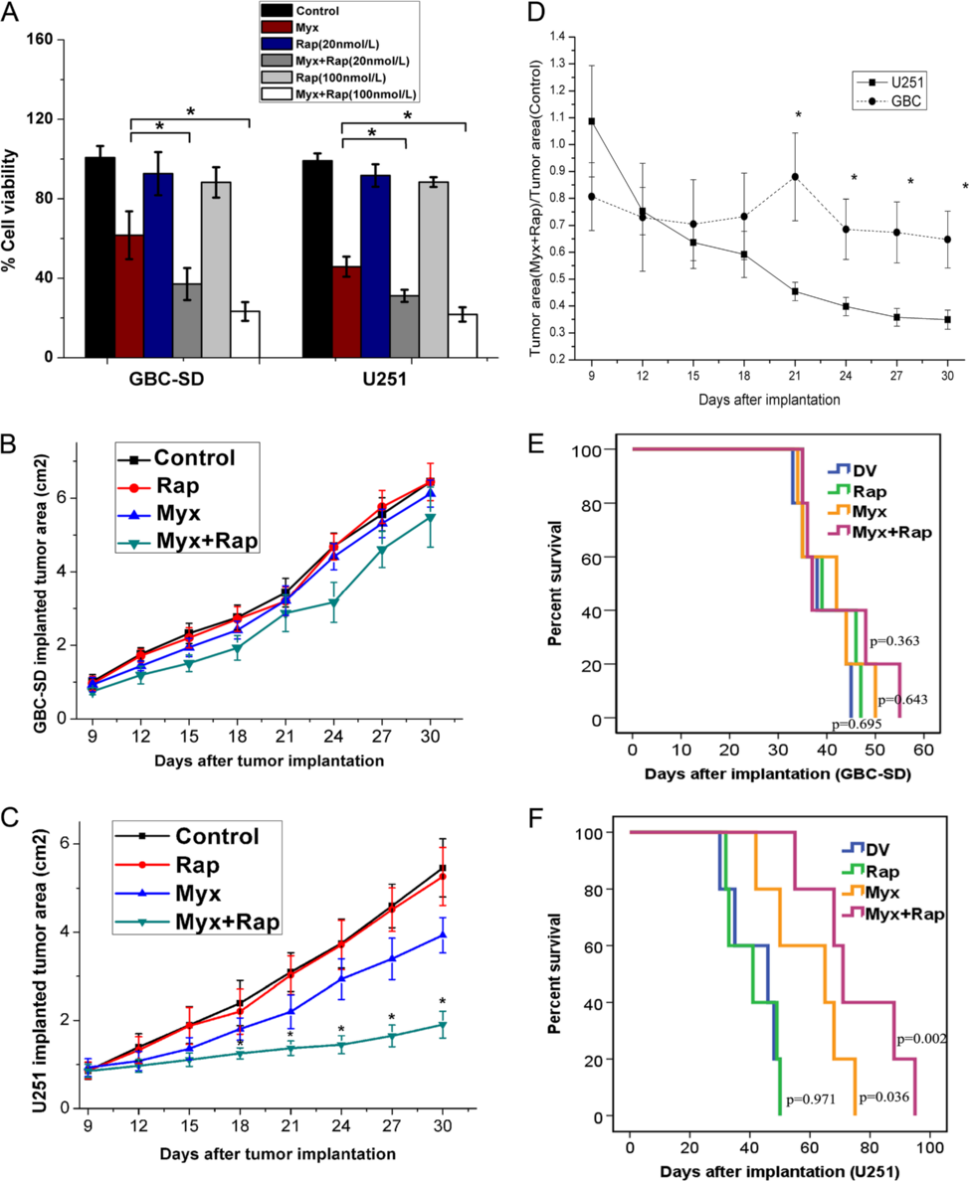
**Effect of rapamycin combined with myxoma virus on human gallbladder cancer cell lines *in vitro *and *in vivo*. A**. The effect of MYXV + Rap on GBC-SD and U251 cells. Cell viability was measured by WST-1 assay 72 h after vMyx-gfp infection in the presence (+) or absence (−) of 20 ng/mL or 100 ng/mL Rap (*, *P* < 0.05 when compared with Myx alone group). **B**, **C**. *In vivo* mouse xenograft model of gallbladder cancer (GBC-SD) or glioma (U251) with UV-inactivated Dead Virus (DV), Rapamycin (Rap), vMyx-gfp (Myx), or Myx + Rap. Tumor areas were evaluated over time by caliper measurement (*, *P* < 0.05 of Myx + Rap vs. Myx). **D**. Relative tumor area was measured to compare the growth pattern of xenografted mice implanted with GBC-SD or U251 (*, *P* < 0.05 of U251 vs. GBC-SD), which was calculated by dividing the tumor area found after combination therapy by that found after control treatment at each time point **E**, **F**. Kaplan-Meier survival analysis of mice treated with DV, Rap, Myx, or Myx + Rap in xenografted mice implanted with GBC-SD or U251.

In terms of oncolysis, MYXV + Rap resulted in significantly more cell death than either treatment alone. With 20 nmol/L Rap in combined therapy, GBC-SD vs. U251 viability was 37.1% vs. 34.2%, respectively (*P* < 0.05); with 100 nmol/L Rap, viability was reduced to nearly identical levels (26.2% vs. 20.7%, respectively; *P* > 0.05) (Figure 2A). Thus, elevating p-Akt levels by Rap improves MYXV-mediated GBC cell oncolysis *in vitro*.

### Pretreatment with rapamycin does not enhance MYXV oncolysis in GBC lines *in vivo*

Since Rap treatment enhanced MYXV-mediated GBC-SD oncolysis *in vitro,* we tested the effects of MYXV + Rap *in vivo*. Day 9 after tumor cell inoculation, mice received DV, MYXV, or MYXV + Rap treatment (Figure 2B,C). MYXV + Rap significantly reduced the area of control U251 tumors starting on day 18 compared to DV (*P* = 0.03), but not that of GBC-SD tumors (*P* > 0.05). To avoid the variations in tumor growth rate, we compared the ratios (fold over control) between the U251 and GBC-SD tumors. Variance in area ratios became significant since day 21 (Figure 2D), suggesting that combination therapy did not have an expected oncolytic effect on GBC-SD bearing tumors *in vivo*. Unlike the MYXV- or MYXV + Rap-mediated host-survival-prolonging effects on U251 bearing mice (Figure 2F), neither treatment prolonged the survival of GBC-SD–bearing mice (Figure 2E).

### Higher expression level of collagen IV in human GBC tumors than in gliomas

**Figure 3:**
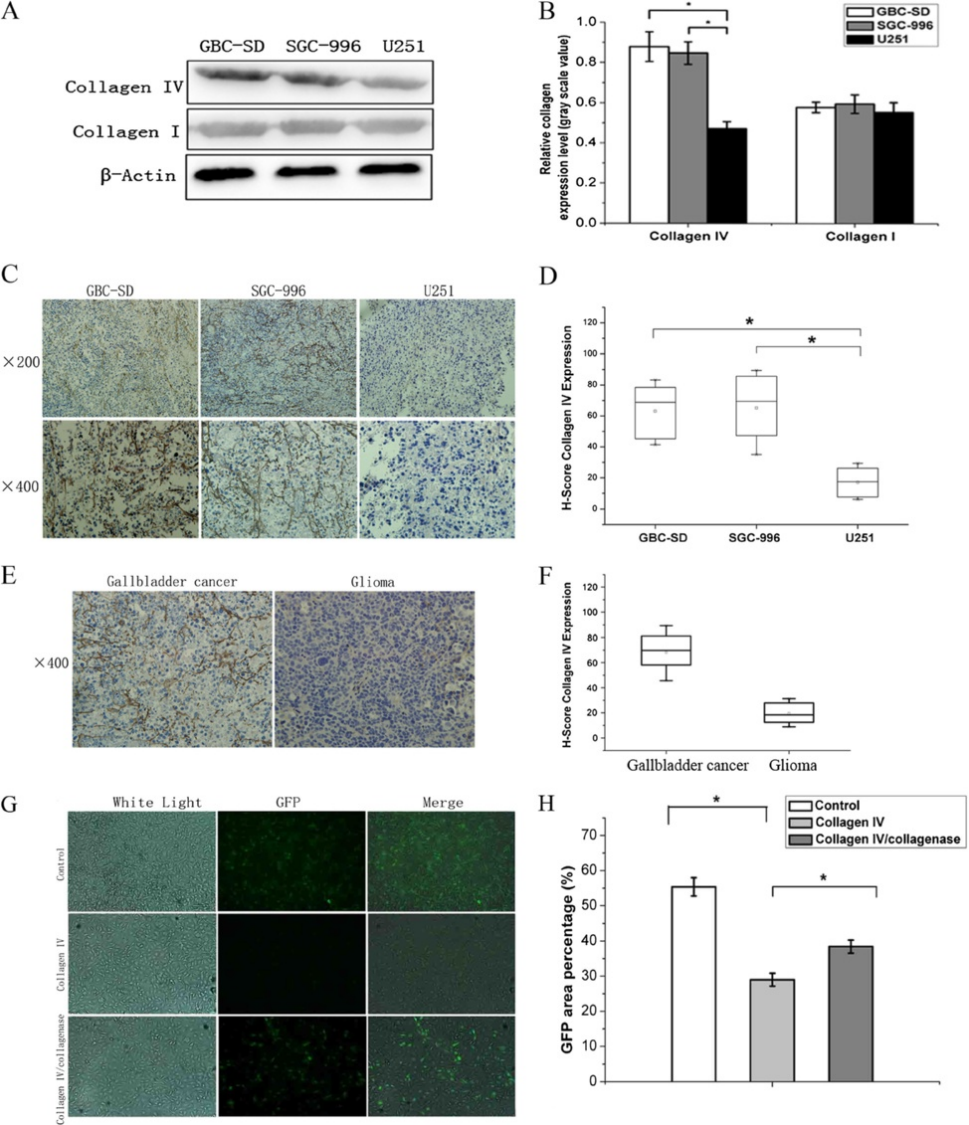
**Collagen IV expression in xenograft tumors and gallbladder cancer tissues, Collagen IV prevents the diffusion of myxoma virus in gallbladder cancer tissues. A**, **B**. **(A)** The western blot of samples from tissue extracted from tumors that were probed with an antibody to collagen I, collagen IV, or ß-actin. **(B)** The histogram represents the relative densitometry levels revealed by the western blot (*, *P* < 0.05 compared with the U251 group). **C**, **D**. **(C)** The immunohistochemistry results showing collagen IV distribution in xenograft tumors formed by implanting GBC-SD, SGC-996, or U251 cells and the **(D)** H-Scores (*, *P* < 0.05 compared with U251 group). **E**, **F**. **(E)** The immunohistochemistry results showing collagen IV distribution in clinical samples of gallbladder cancer or glioma and the **(F)** H-Scores. **G.** Virus diffusion across a collagen matrix barrier. In the top row, the control well allowed free diffusion of the GFP-expressing MYXV. In the second row, collagen IV markedly reduced the proportion of viruses that migrated past the barrier. In the bottom row, the proportion of vMyx-gfp–infected cells was restored to control levels by pre-treatment with collagenase. **H**. The percentage of GFP-positive areas in the examined field (*, *P* < 0.05 compared with the collagen IV group).

Our data demonstrated that oncolytic enhancement effects of Rap was not obvious on GBC-SD cells *in vivo* even though it was significant *in vitro*. Since ECM influences viral particle penetration into tissues and infection of surrounding cells [18], we first explored whether it was an underlying mechanism that prevented MYXVdistribution*.* We examined the levels of major ECM components, collagen I and IV, in GBC-SD, SGC-996, and U251 xenograft tumors by western blot. GBC tumors expressed significantly more collagen IV than U251 (*P* < 0.05) (Figure 3A,B), which was further confirmed by immunohistochemistry (Figure 3C); collagen I, however, was not statistically different among the 3 tumors. The average H-Scores in GBC-SD, SGC-996, and U251 were 63.1, 65.1, and 18.5, respectively (GBC vs. U251, *P* < 0.01) (Figure 3D). Immunohistochemistry of surgical specimens also revealed significantly higher H-Scores in GBC than in primary gliomas (68.1 vs. 19.6, *P* < 0.01) (Additional file 1: Table S1 and Figure 3E,F).

### Collagen IV may hinder myxoma virus dissemination *in situ*

To test whether collagen IV presented a physical barrier to MYXV diffusion, we measured vMyx-gfp diffusion through barrier inserts pre-coated with collagen IV. While 55.36% of the GBC-SD area was GFP-positive in controls, only 17.25% of the area below collagen IV-coated inserts was GFP-positive. Degradation of collagen IV by collagenase partially restored vMyx-gfp diffusion (Figure 3G,H). Thus, collagen IV impedes MYXV dissemination into cells.

To determine whether specific binding occurred between MYXV and collagen IV, vMyx-gfp was placed onto pre-coated or control inserts and aspirated for 12 hours before being applied onto GBC-SDs. Similar percentages of GFP-expressing areas were observed (data not shown), suggesting that collagen IV acts as a physical barrier for, rather than specifically binding to, MYXV.

### Hyaluronan promotes MMP-9 mRNA expression

**Figure 4:**
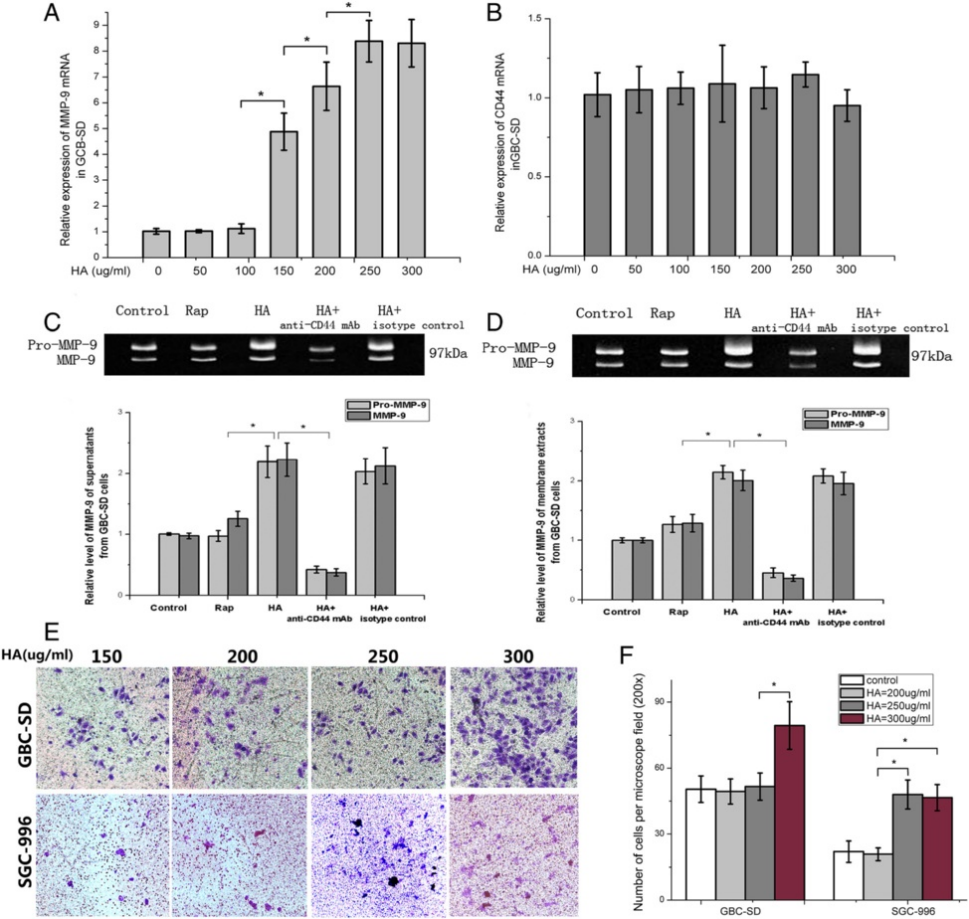
**Gallbladder cancer cell lines express MMP-9 and CD44 mRNA, and hyaluronan treatment increases MMP-9 mRNA expression. A**, **B**. MMP-9 and CD44 mRNA expression in GBC-SD at different HA concentrations (*, *P* < 0.05). **C**. **D**. Gelatin zymogram of MMP-9 and pro-MMP-9 from supernatants and membrane extracts of GBC-SD and their relative activity levels by densitometric scans (*, compared with HA group, p< 0.05). **E**. Crystal violet staining of GBC-SD and SGC-996 invasive cells on the outer surface of the upper chambers following the addition of HA. **F**. Percentage of positive cells per microscope field.

In search of a potential agent that improves the oncolytic effectiveness of MYXV, an initial clue came from earlier studies that suggest that HA enhances MMP-9 expression and Akt activation [16,17]. We first studied whether HA can degrade collagen IV through inducing collagen-degrading MMP-9 expression. Utilizing real-time PCR, HA ≤100 μg/mL did not have an obvious effect on MMP-9 transcript levels, but HA significantly increased MMP-9 transcript levels at 150–250 μg/mL, plateauing at 250 μg/mL (Figure 4A). In contrast, CD44 expression level did not change with increasing dosages of HA (Figure 4B). The results showed that HA induces MMP-9 secretion from GBC tumors, which may function to degrade the surrounding collagen IV.

To determine secreted MMP-9 activity in GBC cells after various treatments, we analyzed MMP-9 activity by gelatin zymogram in supernatants and membrane extracts. In GBC-SD supernatants, MMP-9 activity increased over control after HA (pro–MMP-9: 2.19 ± 0.26, MMP-9: 2.22 ± 0.27, *P* < 0.05), but not Rap treatment (pro–MMP-9: 0.97 ± 0.09, MMP-9: 1.25 ± 0.12, *P >* 0.05), indicating that HA, but not Rap, increased MMP-9 activity (Figure 4C). After incubating HA-treated cells with anti-CD44, both pro–MMP-9 (0.42 ± 0.05) and MMP-9 (0.37 ± 0.06) activity significantly diminished compared to isotype control, suggesting that the HA–CD44 interaction was required for HA-mediated up-regulation of MMP-9 activity. Similar results were observed in membrane extract from GBC-SD. The results of SGC-996 cells were provided in supplemental data (Additional file 2: Figure S1).

### HA–CD44 interaction increases Akt activation and promotes MYXV oncolysis in GBC cells *in vitro*

**Figure 5:**
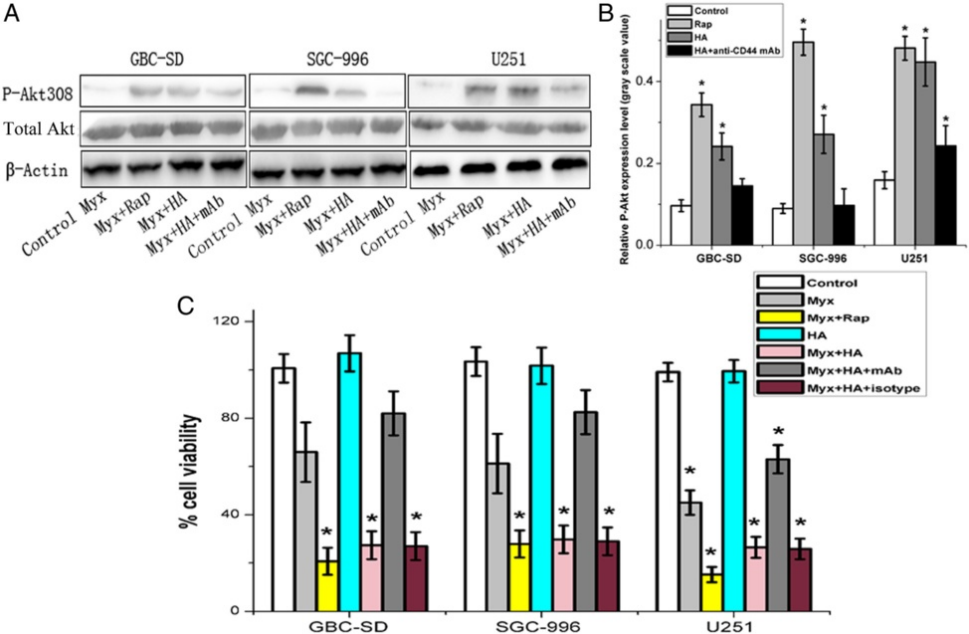
**HA–CD44 interaction increases Akt activation and promotes myxoma virus oncolysis in GBC cells *in vitro*. A**. Western blotting results of p-Akt and total Akt expression in GBC cell lines (GBC-SD, SGC-996) or the glioma cell line (U251) pretreated with MYXV (Myx), Myx + Rap, Myx + HA, or Myx + HA + anti-CD44 mAb. **B**. Relative gray scale of p-Akt values from each group (*, *P* < 0.05 compared with the control group). **C**. Oncolytic effects of MYXV on gallbladder cancer cell lines *in vitro.* GBC-SD, SGC-996, and U251 were infected with control Dead Virus (DV), Myx, Myx + Rap, HA, Myx + HA, Myx + HA + anti-CD44 mAb, or Myx + HA + isotype mAb (*, *P* < 0.05 compared with the control group).

We next investigated whether any functional relationship existed between Akt activation and HA–CD44 interaction in GBC. HA induced significantly higher p-Akt levels than control treatment, which was dependent upon the HA–CD44 interaction (Figure 5A,B). The results suggest that HA increased p-Akt expression, which may correlate with increased susceptibility to MYXV as demonstrated by Wang [7].

To determine whether HA increased MYXV-mediated GBC oncolysis, we examined the cell viability *in vitro.* Although MYXV + HA-mediated oncolysis was less effective than MYXV + Rap, it remained to be superior to other treatments. Thus, HA greatly enhanced MYXV oncolysis of GBC cells *in vitro*, and this was dependent upon the HA–CD44 interaction (Figure 5C).

### Hyaluronan breaks down collagen IV and increases the hydraulic conductivity of GBC cells *in vivo*

**Figure 6:**
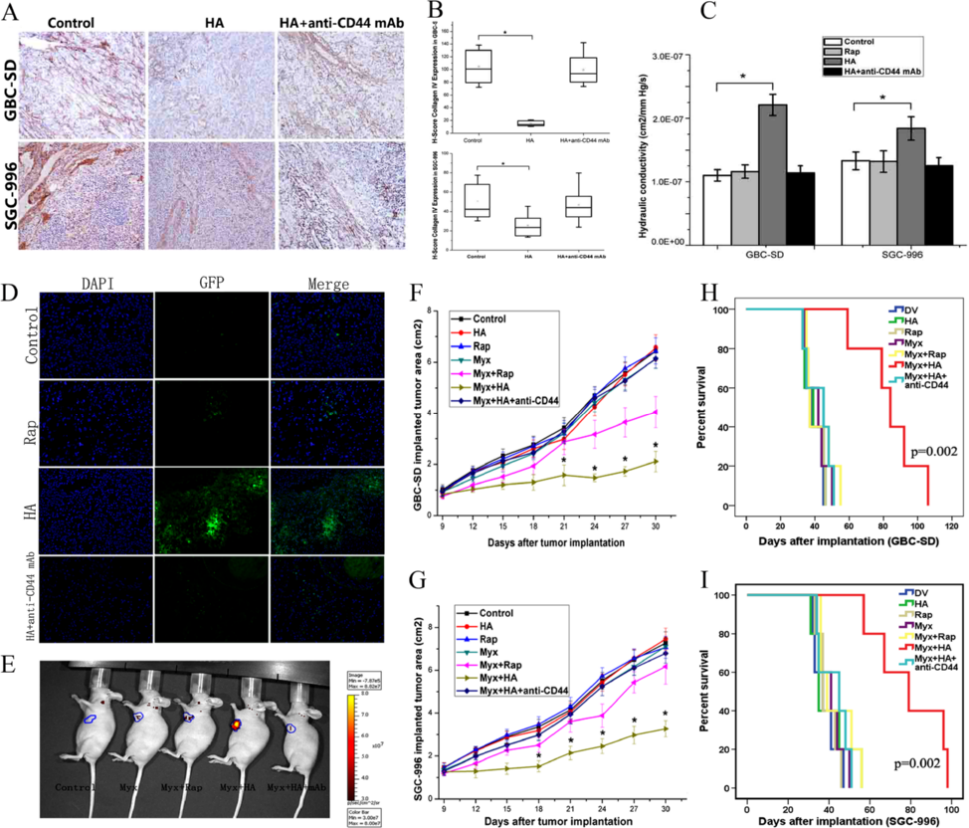
**Hyaluronan breaks down collagen IV and increases the hydraulic conductivity of GBC cells *in vivo.*** Pretreatment with hyaluronan promotes myxoma virus oncolysis in GBC cells and enhances the oncolytic effects of myxoma virus against GBC *in vivo*. **A**. The immunohistochemistry results showing collagen IV distribution in xenograft tumors pretreated with DV (control), HA, or HA + anti-CD44 mAb in GBC-SD and SGC-996 cells. **B**. The H-Scores of collagen IV expression in GBC-SD and SGC-996 xenograft tumors (*, *P* < 0.05 compared with the control group). **C**. The hydraulic conductivity of xenograft tumors pretreated with DV (control), HA, or HA + anti-CD44 mAb in GBC-SD and SGC-996 cells (*, *P* < 0.05 compared with the control group). **D**. Following the addition of DV, Myx + Rap, Myx + HA, or Myx + HA + anti-CD44 mAb, frozen sections of the GBC-SD tumors from GBC-SD tumor-bearing mice were stained with DAPI and examined under fluorescence microscopy. **E**. Representative photographs of GFP-labeled virus in the tumors of GBC-SD tumor-bearing mice. **F**, **G**. *In vivo* treatment of GBC-SD and SGC-996 xenograft tumors with control DV, HA, Rap, Mvx, Myx + Rap, Myx + HA, and Myx + HA + anti-CD44 mAb. Tumor areas were evaluated over time by caliper measurement (*, *P* < 0.05 compared to Myx + Rap). **H**,** I**. Kaplan-Meier survival analysis of xenograft mice implanted with GBC-SD and SGC-996 after treatment with DV, HA, Rap, Myx, Myx + Rap, Myx + HA, and Myx + HA + anti-CD44 mAb (comparing the Myx + HA group with the Myx + Rap group, *P* = 0.002 and 0.002 in GBC-SD and SGC-996, respectively).

As shown above, the tumor-reducing effect of MYXV + Rap was not obvious at GBC-SD tumors *in vivo* (Figure 1B, E)*.* To explore the potential application of MYXV + HA *in vivo*, we first evaluated intratumoral viral infusion in GBC-SD and SGC-996 xenografts utilizing hydraulic conductivity assay. HA, but not Rap, significantly elevated flow conductivity of GBC-SD–forming tumors compared with control (2.21 vs. 1.1, *P* < 0.05), indicating that HA increased GBC tumor permeability for intratumoral liquid flow, which may allow MYXV diffusion. This effect depended upon the HA–CD44 interaction, and a similar trend was observed in SGC-996 (Figure 6C).

To verify that HA increased permeability by degrading collagen IV, western blot and immunohistochemistry showed that HA, but not Rap (data not shown), significantly decreased collagen distribution within tumors and that this effect depended upon the HA–CD44 interaction (Figure 6A,B).

### The safety assessment of HA

Since HA induced collagen IV degradation via enhancing MMP-9 expression, the effects of HA on GBC cell invasiveness should be evaluted. With Transwell invasion assay, the migratory capacity was significantly increased in GBC-SD cells when HA reached 300 μg/mL and in SGC-996 lines 250 μg/mL (Figure 4E,F). The results indicated that HA concentrations between 150–200 μg/mL was able to induce MMP-9 expression while having no obvious effects on the migratory capacity of GBC.

### Hyaluronan treatment promotes MYXV-mediated oncolysis of GBC tumors *in vivo*

We next tested the effects of HA on MYXV-mediated GBC oncolysis *in vivo*. Compared with MYXV alone, Rap pretreatment promoted viral replication, but viral distribution within tumor tissues was confined to small focal areas (Figure 6D). In contrast, viral distribution was more extensive after MYXV + HA, and this depended on the HA–CD44 interaction (Figure 6D). This GFP-labeled viral-load increase in tumors after HA treatment was also detected *in situ* (Figure 6E). The results indicated that HA can greatly promote MYXV distribution.

Finally, we determined whether MYXV + HA could effectively shrink GCB tumors *in vivo* and prolong host survival. GBC tumor areas were significantly reduced following MYXV + HA treatment compared to the other cohorts (Figure 6F,G). Significantly enhanced survival was observed after MYXV + HA treatment in GBC-SD and SGC-996–bearing mice compared to MYXV + Rap treatment (Figure 6H,I). It indicated that MYXV + HA greatly enhances the effectiveness of MYXV-mediated GBC oncolysis *in vivo,* resulting in prolonged survival of the GBC tumor-bearing host.

## Discussion

Gallbladder cancer is an aggressive disease with dismal clinical outcome [1,2]. Oncolytic virotherapy is an innovative alternative to conventional therapies [3], and MYXV not only has an extremely narrow host-species tropism but also can selectively infect and kill many human tumor cells utilizing dysregulated signaling pathways [24]. For example, MYXV treatment of human gliomas (U87 or U251) implanted into immunocompromised mice progressively decreased tumor size, increased host survival, and even completely cured the disease [25,26].

Rap dramatically increases permissiveness of certain type II human tumor cell lines to MYXV [9]. Increased MYXV replication in cells is concomitant with global effects on mTOR signaling and correlates with increased Akt kinase activation [10]. Rap also enhances MYXV oncolysis *in vivo* in a murine xenograft human medulloblastoma model [10]. Here, we demonstrated that two GBC cell lines, GBC-SD and SGC-996, are type II cells (Figure 1). Furthermore, Rap significantly increased p-Akt levels and improved MYXV oncolytic efficiency *in vitro* (Figure 1A). Notably, MYXV-mediated oncolysis of GBC-SD cells was comparable to that of U251 glioma cells in the presence of Rap at 100 ng/mL (73.8% vs. 73.3%). However, in contradictions to previous studies in glioma tumors [9], MYXV + Rap neither significantly reduced tumor area in GBC-SD xenografts nor prolonged host survival compared to MYXV alone (Figure 1B-F).

To explain the discrepancy between MYXV therapy for gliomas and GBCs *in vivo* as well as how MYXV + Rap effectively killed GBCs *in vitro* but not *in vivo*, we hypothesized that tumor-associated ECM may be different between the 2 tumor types. Previous clinical and animal model studies indicate that intratumoral spread of replicating adenovirus *in vivo* can be surprisingly poor compared to viral spread in comparable cell types *in vitro*[27], which may render the virus unable to disseminate within the growing tumor for any clinical benefit [28,29]. Brown et al. found that extracellular collagen hindered diffusive therapeutic-molecule penetration within tumors and that matrix modification alleviated this barrier [18]. Administering collagenase or trypsin to glioma xenografts enhanced infectious adenoviral spread [30]. Furthermore, an MMP-8–expressing adenovirus construct, which effectively degraded collagen I, improved viral spread and oncolysis [31]. The role of tumor-associated collagen in MYXV intratumoral spread, however, has not yet been investigated. We found 2 GBC tumors expressed more collagen IV than U251 glioma tumors *in vivo* (Figure 3A-D). The same outcome was observed when comparing clinical samples from 10 GBC and 5 glioma patients (Figure 3E,F). Thus, increased collagen IV in both xenografts and solid tumors suggested the universality of enhanced collagen distribution in GBC-associated ECM. Functionally, collagen IV significantly blocked MYXV diffusion in diffusion assays, which was restored by collagenase treatment (Figure 3G,H). No binding was detected between collagen IV and MYXV, suggesting that collagen IV likely serves as a physical barrier to prevent viral passage through the membrane and, by inference, within GBC tissues. Thus, the abundant collagen IV distribution within GBCs may account for the poor intratumoral viral spread and suboptimal effect of MYXV + Rap *in vivo*.

To circumvent this collagen IV barrier, we exploited HA––a non-sulfated, unbranched GAG consisting of repeating disaccharide units––as a potential therapeutic approach. HA binding to CD44 not only affects cell adhesion to the matrix but also stimulates several tumor-specific functions. Also, HA–CD44 interactions increase p-Akt levels [32]. Recently, it was shown that HA regulated OPN (a transcriptional target of HA) and that the PI3K/Akt/mTOR pathway upregulated OPN [16]. As expected, we found that the HA–CD44 interaction also mediated and was required for Akt activation in GBC cells. Moreover, considerably enhanced oncolysis by either MYXV + HA or MYXV + Rap was observed in GBCs *in vitro*. Despite the less significant tumor inhibitory effect by MYXV + HA compared to MYXV + Rap in SGC-996 cells, the MYXV + HA regimen was still superior than MYXV alone (Figure 5C).

HA–CD44-mediated enhancement of MMP-9 activity was extensively investigated in other tumors [33-36]. HA–CD44 signaling is thought to stimulate FAK and modulate MMP-9 secretion via Ras-ERK 1/2 signaling [17]. Transcriptional activation of genes containing putative AP-1 and/or NFκB binding sites in their promoter also regulates MMP expression [37]. In our study, HA enhanced both the pro-enzyme and active form of MMP-9 in GBC tumor cell supernatants as well as membrane-bound MMP-9 in membrane extracts (which may regulate pericellular ECM degradation from the tumor cell surface) in a CD44-dependent fashion *in vitro*.

The *in vivo* GBC model best reflects the cellular/extracellular environments influencing tumor formation and susceptibility to oncolytic virotherapy. Our immunohistochemistry analysis showed that HA significantly degraded extracellular collagen IV within tumors in a CD44-dependent manner (Figure 6A,B). Increased hydraulic conductivity confirmed that HA reduced intratumoral fluid flow resistance, helping to rationalize how HA promoted MYXV dissemination. MYXV + HA exhibited superior GBC oncolytic efficiency *in vivo* compared to MYXV + Rap in immunodeficient mice, both in terms of tumor area and overall host survival (Figure 6F-I). However, MYXV + HA did not completely eliminate GBC tumors. It is possible that HA induces inflammatory mediators, such as IFN via a TLR/MyD88-dependent pathway, which may interfere with MYXV proliferation and diffusion [38].

HA–CD44 interactions play important roles in tumor invasion and migration [39]. In our study, we showed that MMP-9 expression rose when HA was above 150, but below 250 ng/mL; in contrast, HA did not increase CD44 expression (Figure 6A-D). The maximal HA safe concentration for GBC-SD and SGC-996 based on the Transwell assay was 250 and 200 ng/mL, respectively. To avoid significantly enhancing tumor-cell migratory capacity, we adopted 200 ng/mL HA *in vitro* and *in vivo*.

We report for the first time that collagen IV is a critical limiting factor impeding MYXV spread in GBC tissue and reveal the synergistic oncolytic effect of MYXV + HA, which may help develop and optimize GBC therapy. However, some caveats still remain. Firstly, MYXV-induced anti-tumor and anti-viral immunity will undoubtedly affect tumor progression in immunocompetent hosts, which will need to be addressed in future studies, and immunocompetent GBC models will be developed to investigate MYXV + HA synergy within an intact immune system. In the second place, the precise mechanism(s) by which HA elevates MMP-9 and p-Akt expression levels are not yet fully understood. Thirdly, anti-CD44 mAb may inhibit GBC tumor growth by hampering apoptosis or angiogenesis [40,41]. Finally, since we based the viral dose on previous reported experience with other tumor types, the ideal MYXV and HA doses against GBC need to be determined.

To conclude, we propose that extracellular tissue collagen IV hinders MYXV dissemination. Moreover, HA–CD44 interaction may elevate oncolytic efficiency not only by activating Akt but also promote viral spread within GBC tissue by degrading collagen IV through MMP-9 secretion, finally converging to enhance the overall MYXV-mediated anti-tumor effect on GBCs *in vivo.*

## Competing interests

We declare that we have no financial and personal relationships with other people or organizations that can inappropriately influence our work, there is no professional or other personal interest of any nature or kind in any product, service and/or company that could be construed as influencing the position presented in the manuscript entitled, “Targeting gallbladder cancer: oncolytic virotherapy with myxoma virus is enhanced by rapamycin *in vitro* and further improved by hyaluronan *in vivo*”.

## Authors’ contributions

WMZ carried out the molecular experiments and drafted the manuscript. GW participated in the molecular experiments and helped to draft the manuscript. MMZ participated in the molecular and animal experiments. CBF carried out immunoassays. QYY and ZMD carried out viral preparation. LXQ helped to draft the manuscript. MG and FP helped to design the study. YY performed the statistical analysis. QZW conceived of the study, and participated in its design and coordination and helped to draft the manuscript. All authors read and approved the final manuscript.

## Supplementary Material

Additional file 1: Table S1 Patient’s characteristics. Click here for file

Additional file 2: Figure S1 A, B. MMP-9 and CD44 mRNA expression in SGC-996 at different HA concentrations (*, *P* < 0.05). C. D. Gelatin zymogram of MMP-9 and pro-MMP-9 from supernatants and membrane extracts of SGC-996 and their relative activity levels by densitometric scans (*, compared with HA group, p< 0.05). Click here for file
