# Supplementary material for: Targeting gallbladder cancer: oncolytic virotherapy with myxoma virus is enhanced by rapamycin in vitro and further improved by hyaluronan in vivo
Source: Mol Cancer. 2014 Apr 13;13:82. doi: 10.1186/1476-4598-13-82 (PMC4021541; doi:10.1186/1476-4598-13-82)
Supplement: Additional file 1: Table S1 — Patient’s characteristics. [file 1476-4598-13-82-S1.docx]

**Additional file 1: Table S1 Patient’s characteristics**

| Code number | Age | Sex | Pathologic diagnosis |
| --- | --- | --- | --- |
| 1 | 42 years | male | Gallbladder cancer |
| 2 | 54 years | male | Gallbladder cancer |
| 3 | 49 years | female | Gallbladder cancer |
| 4 | 56 years | female | Gallbladder cancer |
| 5 | 49 years | female | Gallbladder cancer |
| 6 | 55 years | female | Gallbladder cancer |
| 7 | 59 years | female | Gallbladder cancer |
| 8 | 68 years | female | Gallbladder cancer |
| 9 | 66 years | female | Gallbladder cancer |
| 10 | 59 years | female | Gallbladder cancer |
| 11 | 8 months | female | Teratoid rhabdoid tumor |
| 12 | 5 years | female | Diffuse astrocytoma |
| 13 | 2 years | male | Medulloblastoma |
| 14 | 8 years | male | Glioblastoma |
| 15 | 3 years | male | Pilocytic astrocytoma |
