# Supplementary figures and images for: Targeting gallbladder cancer: oncolytic virotherapy with myxoma virus is enhanced by rapamycin in vitro and further improved by hyaluronan in vivo
Source: Mol Cancer. 2014 Apr 13;13:82. doi: 10.1186/1476-4598-13-82 (PMC4021541; doi:10.1186/1476-4598-13-82)

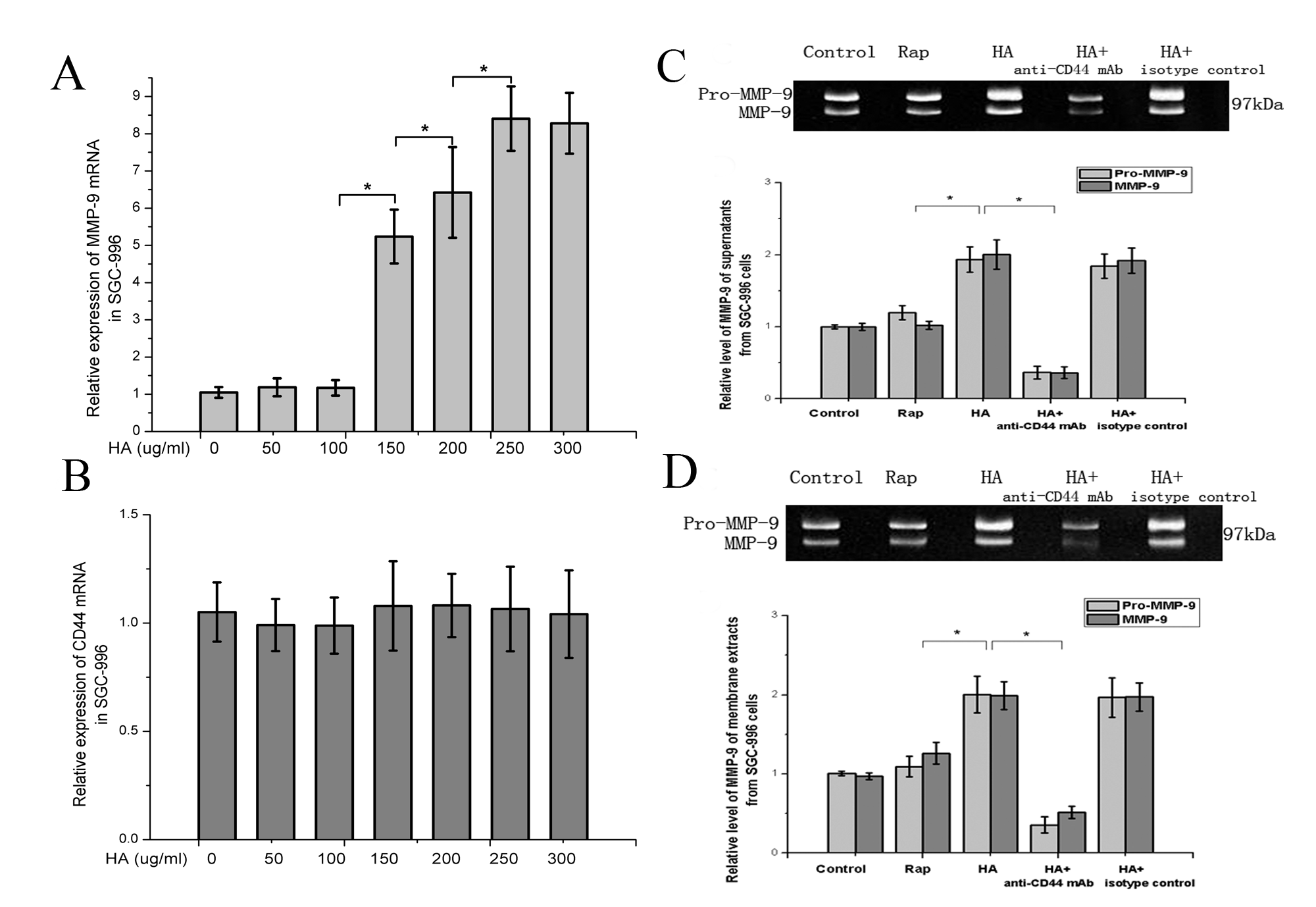

Supplement: Additional file 2: Figure S1 — A, B. MMP-9 and CD44 mRNA expression in SGC-996 at different HA concentrations (*, P < 0.05). C. D. Gelatin zymogram of MMP-9 and pro-MMP-9 from supernatants and membrane extracts of SGC-996 and their relative activity levels by densitometric scans (*, compared with HA group, p< 0.05). [file 1476-4598-13-82-S2.tiff]
